# Transgenic Expression of Human C-Type Lectin Protein CLEC18A Reduces Dengue Virus Type 2 Infectivity in *Aedes aegypti*

**DOI:** 10.3389/fimmu.2021.640367

**Published:** 2021-03-09

**Authors:** Lie Cheng, Wei-Liang Liu, Yun-Ting Tsou, Jian-Chiuan Li, Chia-Hao Chien, Matthew P. Su, Kun-Lin Liu, Ya-Lang Huang, Shih-Cheng Wu, Jih-Jin Tsai, Shie-Liang Hsieh, Chun-Hong Chen

**Affiliations:** ^1^National Institute of Infectious Diseases and Vaccinology, National Health Research Institutes, Miaoli, Taiwan; ^2^Tropical Medicine Center, Kaohsiung Medical University Hospital, Kaohsiung, Taiwan; ^3^National Mosquito-Borne Diseases Control Research Center, National Health Research Institutes, Miaoli, Taiwan; ^4^Institute of Microbiology and Immunology, National Yang Ming Chiao Tung University, Taipei, Taiwan; ^5^Institute of Molecular and Genomic Medicine, National Health Research Institutes, Miaoli, Taiwan; ^6^Department of Biological Science, Nagoya University, Nagoya, Japan; ^7^Genomics Research Center, Academia Sinica, Taipei, Taiwan; ^8^Division of Infectious Diseases, Department of Internal Medicine, Kaohsiung Medical University Hospital, Kaohsiung, Taiwan; ^9^School of Medicine, College of Medicine, Kaohsiung Medical University, Kaohsiung, Taiwan; ^10^Institute of Clinical Medicine, National Yang Ming Chiao Tung University, Taipei, Taiwan; ^11^Department of Medical Research, Taipei Veterans General Hospital, Taipei, Taiwan; ^12^Institute for Cancer Biology and Drug Discovery, Taipei Medical University, Taipei, Taiwan

**Keywords:** transgenic *Aedes aegypti*, dengue virus (DENV), CLEC18A, innate immune pathways, midgut microbiome, mosquito

## Abstract

The C-type lectins, one family of lectins featuring carbohydrate binding domains which participate in a variety of bioprocesses in both humans and mosquitoes, including immune response, are known to target DENV. A human C-type lectin protein CLEC18A in particular shows extensive glycan binding abilities and correlates with type-I interferon expression, making CLEC18A a potential player in innate immune responses to DENV infection; this potential may provide additional regulatory point in improving mosquito immunity. Here, we established for the first time a transgenic *Aedes aegypti* line that expresses human CLEC18A. This expression enhanced the Toll immune pathway responses to DENV infection. Furthermore, viral genome and virus titers were reduced by 70% in the midgut of transgenic mosquitoes. We found significant changes in the composition of the midgut microbiome in CLEC18A expressing mosquitoes, which may result from the Toll pathway enhancement and contribute to DENV inhibition. Transgenic mosquito lines offer a compelling option for studying DENV pathogenesis, and our analyses indicate that modifying the mosquito immune system via expression of a human immune gene can significantly reduce DENV infection.

## Introduction

Approximately half of the world's population is at-risk of infection by dengue virus (DENV) (1). This at-risk population has substantially increased over the past few decades as the major vector of DENV, the *Aedes aegypti* (*A. aegypti*) mosquito, has spread rapidly worldwide. Current methods of vector control have proven inadequate, leading to the development of novel, transgenic mosquito control methodologies (2). This includes the transgenic expression of genes which can modify the mosquito immune system and therefore prevent DENV infection (3). Further refinement of these transgenic lines however requires an improved understanding of this immune system, with our current knowledge of many underlying mechanisms still lacking.

Innate immune systems can be found in vertebrates, invertebrates, and plants, all of which utilize similar mechanisms to regulate basic immune responses against pathogens (4, 5). In mosquitoes, the innate immune system acts as the major protective mechanism against pathogen infection due to the lack of an immunoglobulin-based adaptive immune system (6). Innate immune systems recognize pathogen-associated molecular patterns (PAMPs) in order to identify pathogenic material, which includes many types of biomolecules [e.g., lipopolysaccharides (LPS) (7), nucleic acids (8, 9), microbial peptides or surface proteins (10), and glycans (11, 12)]. PAMPs are recognized by pattern recognition receptors (PRRs), which activate corresponding signaling pathways to initiate various responses. These PRRs include C-type lectin and Toll-like surface receptors, in addition to NOD-like and RIG-like intercellular receptors (13).

Lectins are a class of proteins capable of binding carbohydrates, and mediate a variety of biological processes (14), C-type lectins (Calcium-dependent lectins; CTLs) is one family of lectins (15). CTLs are defined by the presence of the C-type lectin domain (CTLD), which is responsible for carbohydrate binding (16) and has an affinity for various mono- and polysaccharides; particularly in the presence of calcium. CTLs have been found in numerous species, and are reported to have diverse functions (17–20). For example, spleen tyrosine kinase (Syk)-coupled C-type lectin member 5A (CLEC5A) is a PRR for both DENV (21) and Japanese encephalitis virus (JEV), which can induce macrophages and myeloid cells to secrete pre-inflammatory cytokines (22). Furthermore, dendritic cell-specific intercellular adhesion molecule 3-grabbing non-integrin (DC-SIGN) has been reported to be the cell surface receptor for DENV binding and internalization (23–25).

In the human genome, there are at least 57 CTL proteins that can be divided into 17 groups based on their domain architecture (19). *A. aegypti* have 52 CTL proteins which can be classified into four groups based on their size and domain structure (26). Several studies have shown that altering CTL expression in mosquitoes can affect associations between hosts and microbes, such as bacteria (26–29), West Nile virus (WNV) (30), Japanese encephalitis virus (JEV) (31) and DENV (26, 32). Furthermore, interactions between microbes, such as in gut microbiota homeostasis, may also influence the susceptibility of mosquitoes to arboviral infection (33).

In mosquitoes, the C-type lectin and Toll-like receptors are important PRRs for triggering innate immune signaling (34, 35). There are four major innate immune signaling pathways which help mosquitoes combat viral infections, referred to as the Toll, Immune Deficiency (IMD), Janus Kinase/Signal Transducers and Activators of Transcription (JAK/STAT), and RNAi pathways (36, 37). The latter pathway is regarded as the major mechanism by which viral infections are controlled (36). Expression of siRNAs and PIWI-interacting RNAs increase after viral infection (38), and these components can alter viral replication rates (38, 39). Activated Toll, IMD, and JAK/STAT pathways initiate the expression of antimicrobial peptides (AMPs) (36, 37, 40), such as *cecropins, defensins, gambicin, diptericin*, and *attacins*, which combat viral infection via many different mechanisms (41, 42). The relative importance of different CTLs in modulating these pathways remains relatively unexplored, with different CTLs appearing to play significantly different roles (43).

Previous experiments in which immune genes were expressed in *Aedes* mosquitoes, illustrate the potential opportunities available to not only investigate the influence of the CTLs on mosquito immune responses but also for generating novel transgenic lines to use as vector control tools. This includes the introduction of DENV2 RNAi to inhibit the replication of DENV2 (44); expression of specific monoclonal antibody single-chain variable fragments to neutralize several DENV serotypes (45); activation of the JAK/STAT pathway to enhance resistance to DENV infection (46); and knockdown of mosquito *GCTL-3* to reduce DENV2 infection (43).

One particularly promising candidate for such experiments, CLEC18, has been identified as a member of the human C-type lectin protein family, and belongs to the C-type lectin group XV (47). This protein contains one CTLD at the C terminal end, a sperm-coating protein domain [SCP/TAPS, or CAP (cysteine-rich secretory proteins, antigen 5, and pathogenesis-related 1) domain] at the N terminal end, and two EGF (epidermal growth factor)-like domains. CLEC18 has three isoforms (denoted CLEC18A, CLEC18B, and CLEC18C), each of which shows differences in affinity for carbohydrates. For example, CLEC18A has a higher affinity for F3 polysaccharides isolated from medicinal fungi *Ganoderma lucidum* (GLPS-F3), and is calcium-independent (47). CLEC18A is a secretory protein that can be detected in human blood and is negatively correlated with chronic hepatitis B infection in patients (48). Beyond hepatocytes, this isoform can also be secreted from monocytes, dendritic cells, and macrophages, suggesting that CLEC18A may be related to the innate immune system, and therefore may possibly participate in combatting viral infection (47). A mouse model study has demonstrated that the presence of CLEC18A increases type-I interferon expression (49), could leading to reduction in DENV infection and replication. Despite these promising results however, CLEC18A has not yet been expressed in mosquitoes.

Nanostructured electrochemical biosensors are electrochemical impedance spectroscopy-based biosensors (50), by coating bait proteins to the sensor surface and then incubate with target proteins, the interactions between ligand and receptor can be detected by measuring the changes of the impedance signal between the electrode and solution interface. It is a sensitive method for screening weak interaction between molecular, such as protein-glycol conjugation.

Here, we tested the effect of CLEC18A expression on *Aedes aegypti* immune responses to infection. We first established a human CLEC18A transgenic *A. aegypti* mosquito line using CRISPR/cas9. By using ELISA and nanostructured electrochemical biosensors.

## Materials and Methods

### Mosquito Rearing

*Aedes aegypti* (Higgs strain) mosquitoes were used for all experiments. Mosquito eggs were hatched in Reverse osmosis (RO) water. Larvae were then transferred to larger bowls and fed a mixture of yeast powder (Taiwan Sugar Corporation, Taiwan) and lyophilized powder of goose liver (#7573, NTN fishing bait LTD, Taiwan). Pupae were collected and then transferred to cages for adult emergence. Adult mosquitoes were provided with a constant supply of 10% sucrose water and maintained at 28°C and 75% relative humidity under a 12-h light: 12-h dark cycle.

### DENV Maintenance

For the protein binding assay, DENV-2 (PL046) was maintained in C6/36 cells. For all other experiments, DENV-2 (New Guinea C strain, NGC) was passaged using the Vero cell line and stored in a −80°C freezer. Viral titers were determined via plaque assay.

### Plaque Assay

To determine DENV2 titers, 2.5 × 10^5^ BHK cells were seeded into individual wells of a 6-well plate and incubated for 24 h. Virus supernatant was serially diluted (10^−1^-10^−7^) with DMEM and then applied to BHK cells in the 6-well plate. After infection for 2 h, virus supernatant was removed, and cells were washed with PBS. Cells were then covered with DMEM containing 1% methyl cellulose and 2% FBS and incubated for a further 5 days. After this incubation, the DMEM was removed and cells were stained with 1% crystal violet. Plaque numbers were calculated by multiplying the number of plaques counted per well (between 10 and 100) by the relevant dilution factor to obtain Plaque forming units (PFUs) per milliliter.

### Plasmid Construction

Cloning steps described in this section followed procedures outlined for the In-Fusion® HD Cloning system (Clontech, Mountain View, CA). Full primer sequences are listed in Table 1.

**Table 1:** Primers used for plasmid cloning.

**Primer name**	**Sequence**
pAc5.1_3xHA fusion_CLEC18A- F	CATCTCGTACGCATGCTGCATCCAGAGACCTCCCCTGGCCGG
pAc5.1_3xHA fusion_CLEC18A-R	CGTATGGGTACTCGAGGGACCCTGGGCCCCACCGGGAGATGTGCTC
pMOS1_AePUb_fusion_CLEC18A-2xHA-F	GAATTCCGCCAGATCTATGCTGCATCCAGAGACCTCCCCTGGCCGG
pMOS1_AePUb_fusion_CLEC18A-2xHA-R	TGTGACGGATCTCGATTATGCATAGTCCGGGACGTCATAGGGATAGCC
pMOS1-nanos_fusion_CLEC18-CDS-F	ACGAACAAAAGAATTCATCCTAGGATGGCCCTGCACCCCGAAACCTCCC
pMOS1-nanos_fusion_CLEC18-CDS-R	TGTGACGGATCTCGAGTTAGCTTCCTGGTCCCCAACGGCTG
pMOS1_fusion_*Fse*I/*Pst*I-AeCPA-pro-F	GACGAGATCGGCCGGCCCGCCCTGCAGATACATAAACTAGTTTTTGCACA
pMOS1_fusion_*Ec*oRI/*Avr*II-AeCPA-pro-R	ATCCTAGGATGAATTCTCCAACTAACCGATACACACTAACCTGG

To generate the pMOS1_AePUb-CLEC18A-2xHA_3xp3-eGFP plasmids, the coding sequence of CLEC18A was amplified from the pMACS Kk.HA(C)-CLEC18A plasmid (49) by PCR using the primer pair pAc5.1_3xHA_fusion_CLEC18A-F and pAc5.1_3xHA_fusion_CLEC18A-R. PCR-amplified DNA fragments were inserted into the *Sph*I/*Xho*I site of the pAePUb_3xHA vector to create a pAePUb_CLEC18A_3xHA transition plasmid. The transition plasmid pAePUb_CLEC18A_3xHA was then used as a template to amplify the CLEC18A-2xHA DNA fragment via PCR using the primer pair pMOS1_AePUb_fusion_CLEC18A-2xHA-F and pMOS1_AePUb_fusion_CLEC18A-2xHA-R. The CLEC18A-2xHA PCR product was subcloned into the *Bgl*II/*Xho*I sites of the pMOS1-AePUb_Den3-4miR_3xp3-eGFP vector to create pMOS1_AePUb-CLEC18A-2xHA_3xp3-eGFP constructs, which were then used as donor plasmids for embryo microinjection.

### Generation of Transgenic Mosquitoes

Donor and helper plasmids were mixed in injection buffer (5 mM KCl and 0.1 mM NaH_2_PO_4_, pH 6.8) at concentrations of 500 and 300 ng/μl, respectively. This DNA mixture was then microinjected into pre-blastoderm stage embryos as described previously (51). G_0_ male and female transgenic mosquitoes were mated with wild-type at a ratio of 1:3. G_1_ larvae were screened for eGFP reporter expression and those lacking eGFP expression were discarded. G_1_ mosquitoes with positive reporter expression were then self-crossed at a ratio of one male to one female. Eggs laid by G_1_ females were collected, and G_1_ parent genotypes were sequenced in order to identify progeny genotypes. After genotype analysis, 3 transgenic lines were obtained.

### Infection of Mosquitoes With DENV2

To infect DENV to mosquitoes artificially, mouse blood was prepared followed Wu et al. (33). Fresh mouse blood was centrifuged at 4°C for 10 min to separate plasma and blood cell components. The plasma was then heat treated at 55°C for 1 h. Blood cells were washed three times with PBS. Plasma and blood cell components were then mixed. Virus supernatant from the DENV2 infected Vero cell line was combined with treated mouse blood to yield 10^7^ PFU/ml virus blood. This infected blood was then provided to mosquitoes via an artificial membrane feeder for 30 min. Successfully blood-fed mosquitoes were visually identified and maintained separately from non-blood fed mosquitoes. To infect mosquito with DENV2 intrathoracically, 400 PFU of DENV2 was injected into the mosquito thorax using a micro-injector (Nanoject II, Drummond Scientific Company). Injected mosquitoes were then maintained in regular conditions.

### RNA Extraction, Reverse Transcription, and qPCR

Midguts of mosquitoes that consumed the infected blood meal (BM) were dissected at 1, 3, and 7 days post-BM (or infection). Total RNA was extracted by TRI reagent (Merck) following the manufacturer's protocol. Extracted total RNA was immediately reverse transcribed using SuperScript III Reverse Transcriptase (Thermo Fisher Scientific). This involved treating 2 μg total RNA with DNase I, Amplification Grade (Thermo Fisher Scientific), followed by first-strand cDNA synthesis. cDNA synthesized from 10 ng total RNA was used as a sample for the relative real-time PCR analysis of CTL expression. Real-time PCR was performed using KAPA SYBR FAST ROX Low (KAPA biosystems) on a ViiA 7 real-time PCR system (Thermo Fisher Scientific). Three biological replicates were performed, with the *A. aegypti* S7 ribosomal protein (RPS7; AAEL009496) expression level used for normalization. All primers used are listed in Table 2.

**Table 2:** Primers used for real-time PCR analysis of innate immune pathways.

**Name**	**Sense primer sequence**	**Antisense primer sequence**	**References**
Toll	TTGGATGGAAACGAGATATCAGAA	CTTCCTGAATCTCGGTCAACTTG	(52)
Rel1A	TGGTGGTGGTGTCCTGCGTAAC	CTGCCTGGCGTGACCGTATCC	(52)
Cactus	AGACAGCCGCACCTTCGATTCC	CGCTTCGGTAGCCTCGTGGATC	(42)
Imd	TCGTCAAACTCGGTTTTCCT	TGGCGGAGTTGAAGGTAAAG	(53)
Rel2	GTTCCCGGATTAGCTACTGTG	CCGTTCCGTTTGCAATGAC	(53)
Caspar	GAATCCGAGCGAGCCGATGC	CGTAGTCCAGCGTTGTGAGGTC	(42)
Vago	CAGTAGCATTTGCCGGTCAGA	CGATGTTGGATCGTAGCACTTC	(52)
Dome	AAACGGTGGCAAAATGAACT	CATACAGCCGGCTTTCTTCT	(52)
Hop	GCTGGTAGTAATGCTTCGAGTGAGT	GCCGGTGCTGTAATGACTAGAA	(52)
STAT	CACACAAAAAGGACGAAGCA	TCCAGTTCCCCTAAAGCTCA	(52)
PIAS	GCTGCAACGCATGAAAACTA	CAGACGGGACAGTTCCAAGT	(43)
Dicer2	CGCTCGGCTTTGGTGAAT	TGCCGACTCTGCCAGGAT	(52)
R2D2	GAAAGGGCTGAGCGATATTGA	CCCGCACTTCGGTCACTTTA	(52)
DefA	CTATCAGGCTGCCGTGGAG	CAATGAGCAGCACAAGCACTATC	(53)
DefC	CTTTGTTTGATGAACTTCCGGAG	GAACCCACTCAGCAGATCGC	(53)
DefD	GGCGTTGGTGATAGTGCTTG	CACACCTTCTTGGAGTTGCAG	(53)
CecN	CGGCAAGAAATTGGAAAAAGTC	GAATCGATCATCCTAGGGCC	(53)
CLEC18A	AGCCAGGATGAAATGTCAGAGGAA	GGTGAGCCCGATCCAGAAGTTC	
DENV-2	GATCACAGGGAACATGTCCTTTA	TAGTAGCGCCCACCATAACC	
RPS7	CAACAGCAAGAAGGCTATCG	TTGCCGGAGAACTTCTTTTC	
ACT	CGTTCGTGACATCAAGGAAA	GAACGATGGCTGGAAGAGAG	(54)
α-tubulin	CTGCTTCAAAATGCGTGAAT	GGTTCCAGATCGACGAAA	(54)

### Preparation of Recombinant Proteins for Binding Assay

In order to produce IgG-like sensing probes for the protein binding assay, the Fc region of human IgG1 was fused with the ECD domain of Dectin-1, DC-SIGN, and CLEC5A (Dectin-1.Fc, DC-SIGN.Fc, and CLEC5A.Fc), or the CTLD domain of CLEC18A, CLEC18A-1, CLEC18B, and CLEC18C (CLEC18A.Fc, CLEC18A-1.Fc, CLEC18B.Fc, and CLEC18C.Fc) as detailed in previous studies (21, 47).

Recombinant protein constructs were expressed using the FreeStyle™ 293 expression system. In total, 30 μg of constructed DNA and 60 μl of 293fectin were transfected into 3 × 10^7^ 293F cells. Cells were incubated for 3–5 days, before the supernatant was harvested. Recombinant sensing probes were purified using a Protein A column (GE Healthcare).

To produce tagged dengue prM-E, FLAG-tagged prM-E constructs were transfected and expressed in 293T cells. Following cell lysis, prM-E-FLAG recombinant protein was purified using anti-FLAG Ab conjugated resin.

### Enzyme-Linked Immunoassay

To analyze the affinity of C-type lectins to prM-E, 2 μg of prM-E-FLAG recombinant protein was immobilized in each well of a 96-well plate (Corning) at 4°C overnight. After incubation, wells were washed twice with 0.05% Tween-20 in TBS (TBST) and then blocked with 2% BSA/TBST at room temperature for 1 h. IgG-like sensing probes (2 μg/ml) were incubated with prM-E-FLAG in calcium binding buffer (2 mM CaCl_2_/2 mM MgCl_2_ in 1% BSA/TBST) or calcium-free binding buffer (5 mM EDTA in 1% BSA/TBST) for 1 h. After washing with TBST, 100 μl of peroxidase conjugated goat anti-human IgG antibody (diluted 1/5,000 in 1% BSA/TBST, Jackson ImmunoResearch) was added to each well and samples were incubated at room temperature for 1 h before a further TBST wash. Following this, 100 μl tetramethylbenzidine substrate (BD Bioscience) was added for 20 min before the reaction was halted by adding 50 μl of 2 N H_2_SO_4_. Protein affinity was analyzed via optical density (OD) calculation at 450/570 nm using an ELISA reader (TECAN).

### Nanostructured Electrochemical Biosensor

The binding affinity of CLEC18 isoforms to dengue virus was tested as described previously (50). A thin layer of anodic aluminum oxide (AAO) on a glass plate was treated with a conventional anodization process (0.3 M phosphoric acid at 90 V and 0°C) to increase reaction surface area. The treated AAO was then washed with CuCl_2_·HCl. The AAO barrier-layer was treated with 30% phosphoric acid for 30 min before a 10 nm gold thin film was layered onto the treated surface to be used as an electrode for the sensor chip.

To analyze the affinity of the C-type lectins for dengue virus, 20 μl of 10 mM 11-mercaptoundecanoic acid (11-MUA) was added to the surface for 10 min, followed by treatment with a 20 μl mixture of 50 mM N-hydroxysuccinimide (NHS) and 100 mM 1-ethyl-3-(3-dimethylaminopropyl)-carbodiimide (EDC). IgG-like sensing probes (0.012 μg/μl) and human IgG1 (0.02 μg/μl) were immobilized on the treated sensor surface for 30 min and then blocked with culture medium for 45 min. Chips were then incubated with 9.5 × 10^7^ PFU/ml of DENV2 (PL046) for 30 min. Electrical resistance before and after dengue virus incubation was measured using a SP-150 potentiostat (Bio-Logic).

### Midgut Microbiome Analysis

Midguts were collected from 25 adult control or CLEC18A transgenic mosquitoes aged 4–5 days using an aseptic process. Samples were then sent for midgut microbiome analysis at Biotools Co., Ltd (New Taipei City, Taiwan). Briefly, bacterial 16S rRNA V3-V4 hypervariable region amplicons in each sample were used as the template for secondary PCR. The products were then added to Illumina sequencing adapters (Illumina) at both ends by using the Nextera XT Index Kit with dual indices (indexed PCR product). Equal amounts of indexed PCR products were then mixed to generate the sequencing library. After purification, libraries were subjected to next generation sequencing (300 bp pair-end reads) using a MiSeq system (Illumina, San Diego, CA). Data were then used for bioinformatic analysis.

### Statistical Analysis

All analyses were performed using GraphPad Prism. Statistical testing comprised of the use of student's *t*-tests, with *p* < 0.05 considered as statistically significant for all comparisons apart from the binding assay of CLEC18 isoforms and DENV viral proteins. Here, we utilized a Bonferroni correction to account for multiple comparisons, meaning that *p* < 0.0125 was considered as statistically significant in this case.

## Results

### Human CLEC18A Associates With Dengue prM-E and Virus Particles

C-type lectins act as important components of many innate immune systems, and are known to act as PRRs of several viruses (including DENV). In mice, transgenic expression of human CLEC18A enhances BMDM IFN-α/β expression following DENV exposure (49). CLEC18A may thus interact with DENV directly to enhance cellular response levels. To investigate whether the human C-type lectin CLEC18 demonstrates direct interactions with DENV proteins, three isoforms of CLEC18 were assayed for affinity with the dengue prM-E protein.

**Figure 1:**
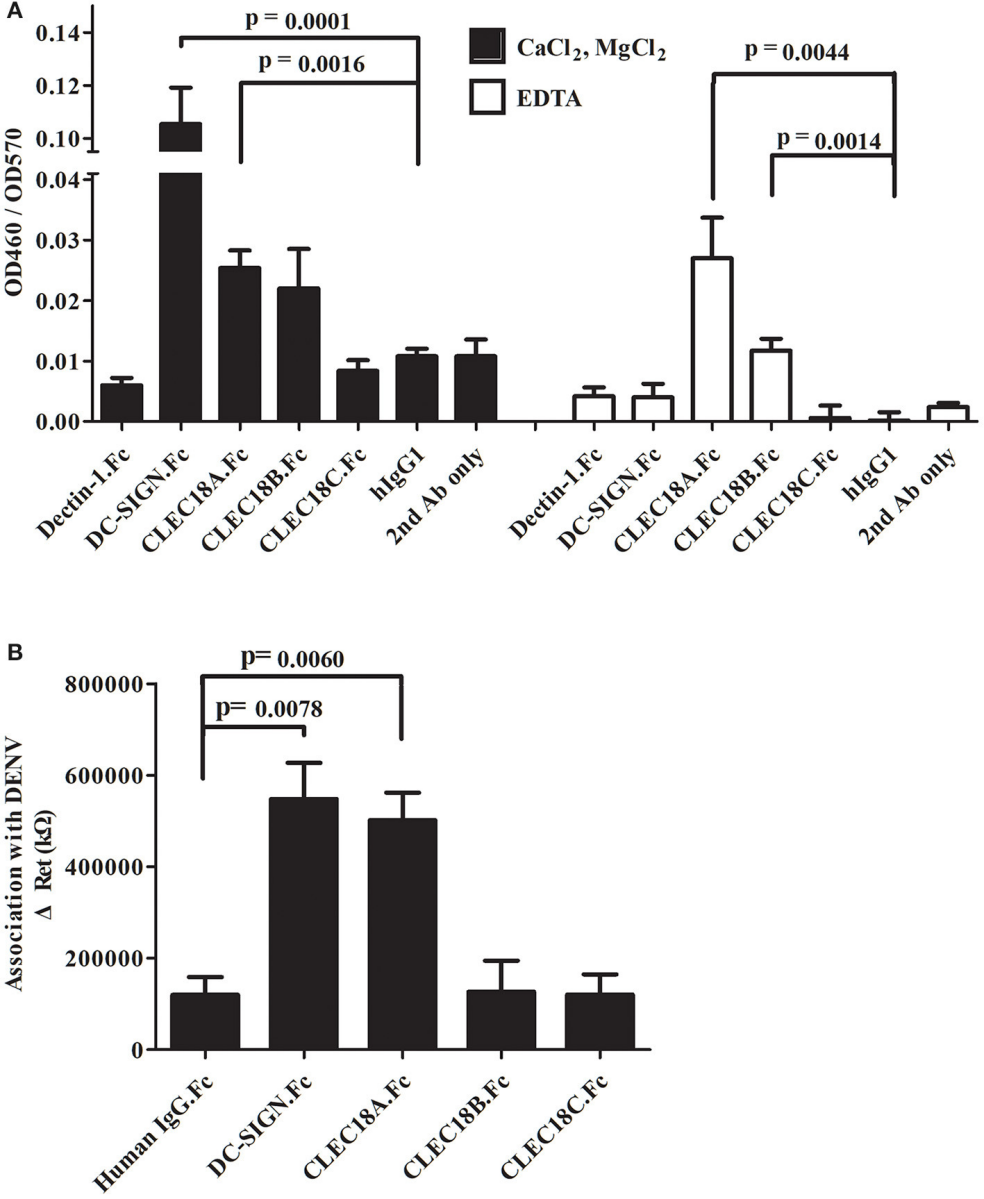
CLEC18A interacts with DENV prM-E in the presence and absence of calcium. **(A)** Purified FLAG-tagged dengue prM-E recombinant proteins were coated on 96-well plates and then incubated with purified human IgG1 Fc domains fused to the ECD domain of Dectin-1 and DC-SIGN (Dectin-1.Fc and DCSIGN.Fc) or CTLD domains of CLEC18A, CLEC18A-1, CLEC18B, and CLEC18C (CLEC18A.Fc, CLEC18A-1.Fc, CLEC18B.Fc, and CLEC18C.Fc) in CaCl_2_/MgCl_2_ or EDTA binding buffer. Bound Fc-fused proteins were detected with anti-hIgG-HRP and TMB. Human IgG1, Dectin-1.Fc, and secondary antibodies were used as negative controls. Since DCSIGN.Fc has been reported to bind with prM-E in the presence of calcium (21), it was used as a positive control. **(B)** Protein probes (DCSIGN.Fc, CLEC5A.Fc, CLEC18A.Fc, CLEC18A-1.Fc, CLEC18B.Fc, and CLEC18C.Fc) were fixed on the surface of nanostructured electrochemical biosensors and then incubated with dengue virus type 2 (PL046). The resistance values produced by the binding of probe and virus were measured and analyzed. The *p*-value represents significant differences between the experiment group and human IgG1 control. Significant *p*-value threshold were corrected by Bonferroni correction using four sets of comparison (DC-SIGN, CLEC18A, CLEC18B, and CLEC18C to hIgG), the corrected *p*-value threshold was *p* < 0.0125.

The CTLD domains of CLEC18A, CLEC18B, and CLEC18C were fused with the human IgG1 Fc domain (here denoted CLEC18A.Fc, CLEC18B.Fc, and CLEC18C.Fc) and then incubated with dengue prM-E in calcium-containing (CaCL_2_/MgCl_2_) or calcium-free (EDTA) buffer. Analysis of ELISA results showed that CLEC18A associated with prM-E in the presence and absence of calcium. CLEC18B showed significant association with prM-E in the absence of calcium. CLEC18C did not have any association with prM-E (Figure 1A). To further confirm the association between CLEC18A and dengue viral protein, CLEC18A.Fc, CLEC18B.Fc, and CLEC18C.Fc were fixed on the treated surfaces of the nanostructured electrochemical biosensor and then incubated with dengue virus. CLEC18A showed a significant interaction with the dengue viral protein (*t*-test; *p* = 0.0264), but CLEC18B and CLEC18C did not (Figure 1B).

### Human CLEC18A Expressed in *Aedes aegypti* Driven by the *Aedes* Polyubiquitin Promoter Interacted With DENV Within Infected Mosquito Cells

The results shown in Figure 1 demonstrate the affinity of CLEC18A to DENV viral proteins, and suggest that CLEC18A may act as a PRR in the human innate immune system. This also makes CLEC18A of interest from a mosquito immune prespective; however, protein BLAST analysis revealed no orthologs of human CLEC18A in the *A. aegypti* peptide database (AaegL5.2 geneset in Vectorbase).

**Figure 2:**
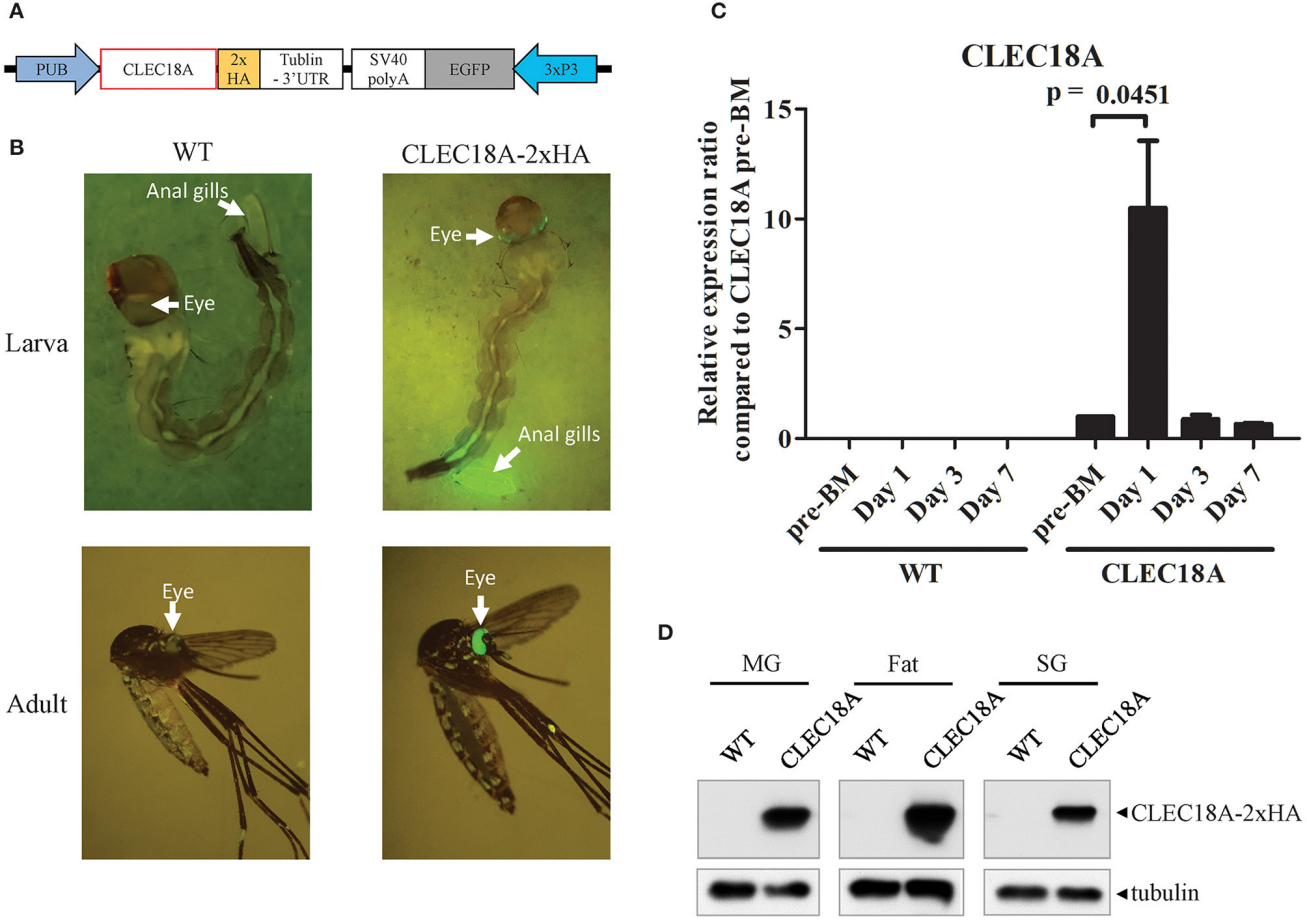
CLEC18A overexpression under the control of the AePub promoter in transgenic *A. aegypti*. **(A)** Schematic representation of the expression construct map used to express CLEC18A-2xHA in transgenic mosquitoes. **(B)** eGFP marker expression in larva or adult of CLEC18A-2xHA transgenic line **(C)** Transcription of Pub promoter-driven CLEC18A-2xHA at different time points post blood meal in transgenic mosquitoes. Bars represent the mean relative fold change of CLEC18A-2xHA expression compared to the pre-BM timepoint of CLEC18A-2xHA according to qPCR. Error bars represent standard error of mean (SEM) **(D)** CLEC18A-2xHA expression in the midgut (MG), fat, and salivary gland (SG) of transgenic mosquitoes by immunoblotting. CLEC18A-2xHA was detected via anti-HA antibody to detect the HA tagged CLEC18A construct. PUB, Poly-Ubiquitin promoter; 3xP3, eye-specific expression promoter.

We thus generated transgenic *A. aegypti* lines expressing human CLEC18A to test whether the presence of CLEC18A could enhance the ability of the mosquito innate immune system to control the replication of DENV. We engineered a pMOS1 vector comprising a mariner transposon cassette (55), which included the CLEC18A cDNA sequence flanking a 2xHA tag under the control of the *A. aegypti* polyubiquitin (AePub) promoter (56) (Figure 2A) to constitutively express CLEC18A-2xHA. This engineered cassette also contained an eye-specific 3xP3 promoter driving expression of eGFP as a transgenesis marker (Figure 2B).

Following embryo microinjection, three separate transgenic lines were identified by DNA sequencing. We selected one of these lines due to a significantly high level of eGFP expression. To confirm CLEC18A-2xHA expression in these transgenic mosquitoes, we performed qPCR analyses of the midguts of blood-fed female CLEC18A transgenic and WT mosquitoes. We identified CLEC18A-2xHA expression only in mutant mosquitoes (Figure 2C). In these mosquitoes, CLEC18A-2xHA was expressed at basal levels; however, these levels were found to significantly increase 1 day after a blood meal (BM) (Figure 2C).

To further confirm CLEC18A-2xHA expression in the tissues of transgenic mosquitoes, we dissected the midguts, fat, and salivary glands of 7 days post-BM mosquitoes for immunoblotting with a HA tag antibody. Expression of transgenic CLEC18A-2xHA was detected in all three tissues (Figure 2D). The expression level of CLEC18A-2xHA was found to be highest in fat tissue, as fat is the major mosquito immune organ.

**Figure 3:**
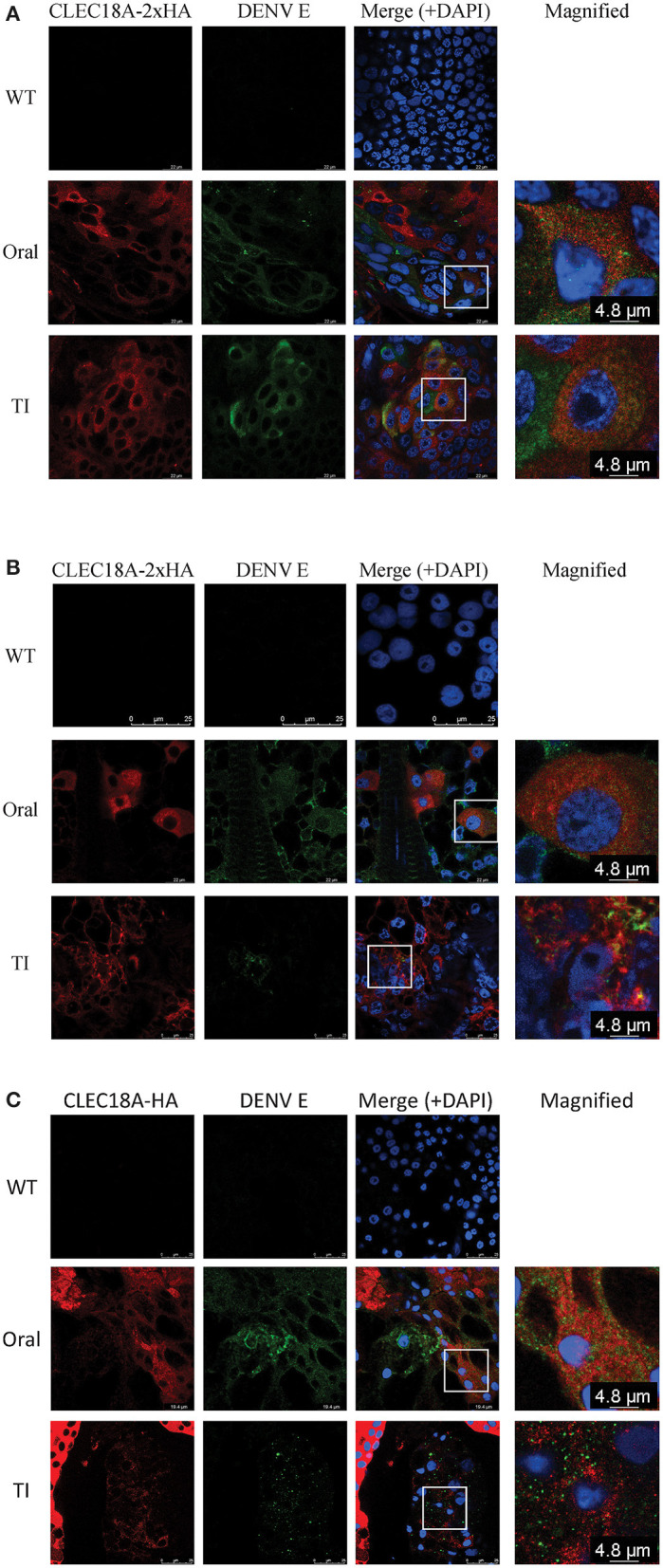
CLEC18A-2xHA is found at the same location as DENV E protein in cells from transgenic *A. aegypti*. CLEC18A-2xHA transgenic mosquitoes were infected with DENV orally or intrathoracically for 7 days, after which midguts (MGs) **(A)**, fat **(B)**, and salivary glands (SGs) **(C)** were harvested and dissected. Dissected tissues were immunostained with anti-HA (Red) and anti-E (Green) antibodies. The rectangular area present in merged images is magnified in the last column.

As we found that CLEC18A was able to interact with DENV (Figure 1), we next investigated whether CLEC18A-2xHA was associated with the DENV viral proteins within infected transgenic mosquito cells. We infected transgenic mosquitoes with DENV either orally or intrathoracically and incubated for 7 days. The midguts, fat, and salivary glands were dissected for imaging analysis. Expression of CLEC18A-2xHA was identified within cells from all tissues, as the Pub promoter is a ubiquitous expression promoter. In the cell expressed both CLEC18A-2xHA and DENV viral protein, part of the E protein and CLEC18A-2xHA can be found in the same location (Figures 3A–C), implying that CLEC18A-2xHA associates with DENV in the infected mosquito cells. However, the expression level of CLEC18A-2xHA differed between cells, resulted in weak signal in some of the cells.

### CLEC18A Expression Results in a Decrease in DENV Viral Titer in Infected Mosquitoes

**Figure 4:**
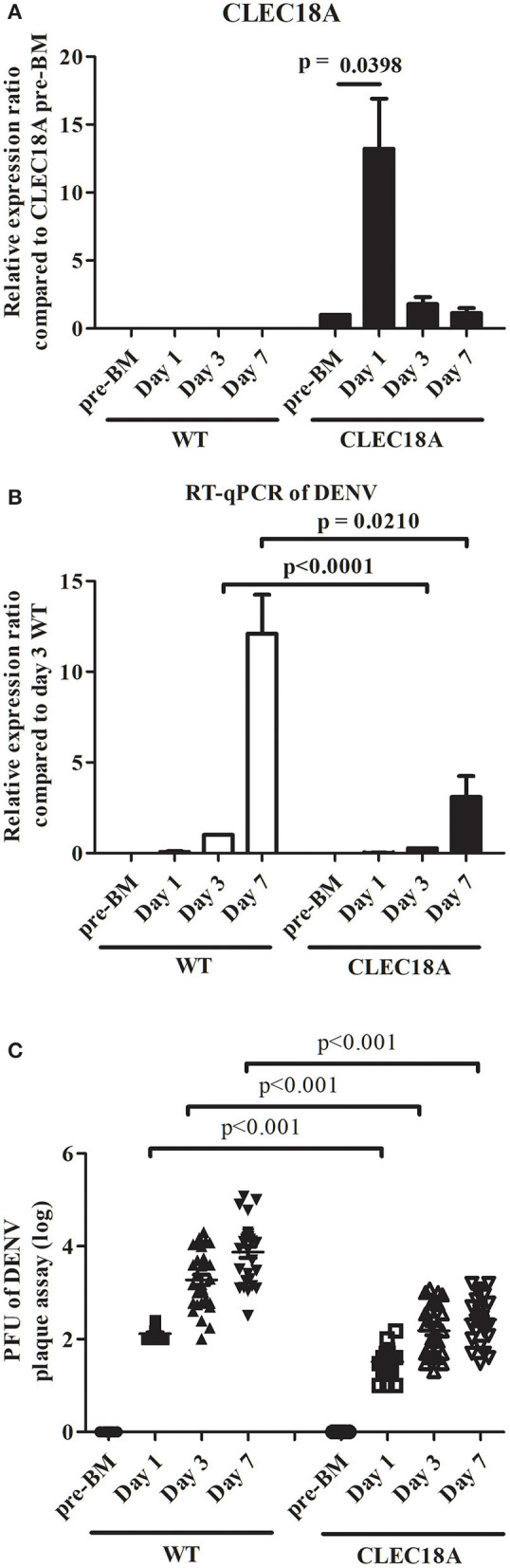
Dengue virus replication and titer in orally infected CLEC18A-2xHA transgenic *A. aegypti* is reduced compared to WT. WT and CLEC18A-2xHA transgenic mosquitoes were fed 10^7^ PFU of DENV2 via artificial blood meal exposure. Midguts at pre-BM, 1 day, and 3 days post-infection were dissected and used to extract RNA for gene expression analysis by qPCR. **(A)** Mean relative expression of CLEC18A in transgenic mosquitoes. CLEC18A pre-BM expression of the CLEC18A samples were set as baseline. Error bars represent standard error of mean (SEM) **(B)** Relative amount of dengue viral genome in infected WT and CLEC18A transgenic mosquitoes. The ratio of the amount of dengue viral genomes for each sample was compared to the sample at 3 days post-infection in WT mosquitoes. **(C)** DENV2 titer of individual WT and CLEC18A transgenic mosquito midguts.

To address the influence of CLEC18A in determining DENV infection outcome, mutant CLEC18A-2xHA mosquitoes (Figure 4A) were infected with DENV2 (NGC strain) via infected BMs. We then used qPCR and plaque assays to investigate DENV replication. At 3 and 7 days after infection, qPCR analysis showed 73 and 74% reductions in the viral genome of the infected CLEC18A-2xHA line (*t*-test; *p* < 0.001; Figure 4B). Plaque assays also indicated 71, 66, and 62% reductions in the viral titers of the CLEC18A-2xHA line at 1, 3, and 7 days post-infection, respectively (*t*-test; *p* < 0.001; Figure 4C).

### CLEC18A Activates Innate Immunity Pathways and AMPs in Transgenic *Aedes aegypti*

DENV replication and production was significantly downregulated in CLEC18A-2xHA transgenic mosquitoes, implying that CLEC18A contributes to the regulation of innate immunity. We therefore investigated if CLEC18A-2xHA expression alters mosquito immune responses to DENV infection. We orally infected WT and CLEC18A-2xHA transgenic mosquitoes using infected BMs, and used qPCR to test for differences in gene expression levels (using RPS7 as a reference) for several components of the Toll, IMD, JAK/STAT, and RNAi immune response pathways at several different time points: pre-BM, and 1, 3, and 7 days post-infection, to facilitate comparisons with the viral load experiment.

**Figure 5:**
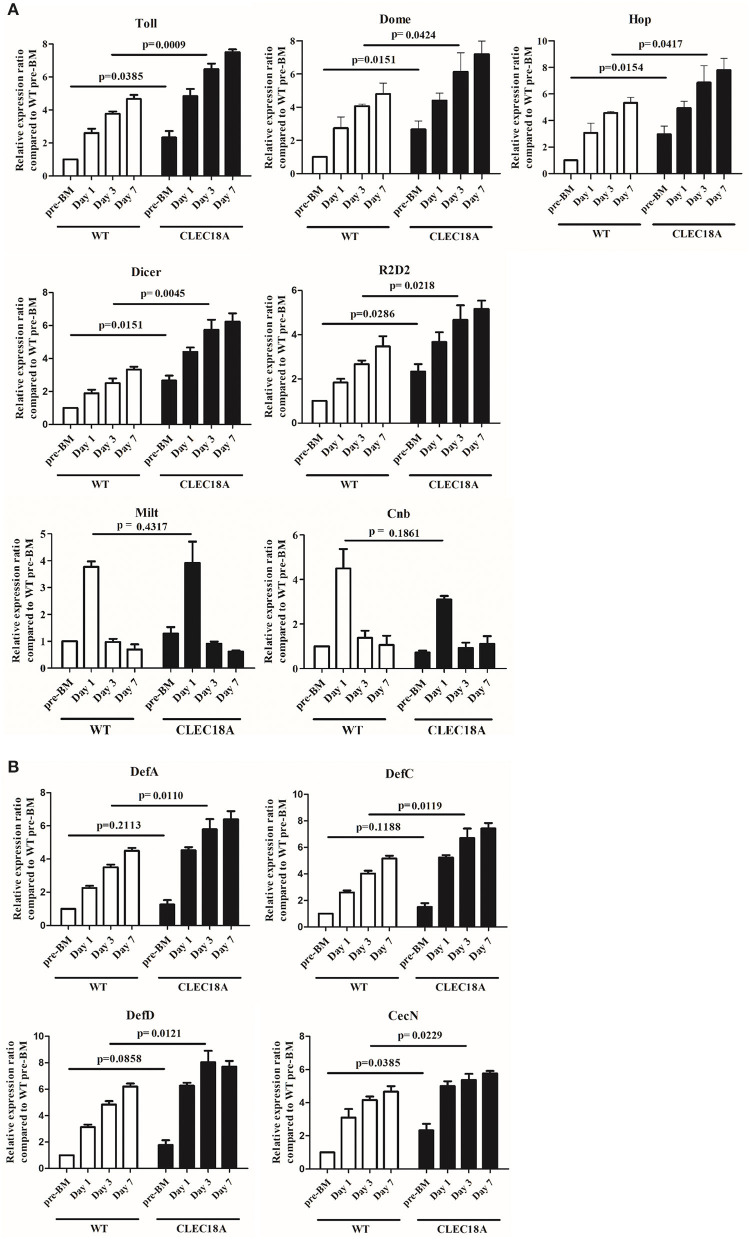
Activation of immune pathways was relatively higher in the CLEC18A-2xHA transgenic line after oral challenge with DENV2. WT and CLEC18A-2xHA transgenic line were infected with DENV via exposure to an artificial blood meal. The midguts of pre-BM, 1, 3, and 7 days post-infection mosquitoes were analyzed to determine the relative expression of components from the immune pathways with qPCR analysis, using RPS7 as a reference gene. **(A)** Expression levels of Toll (Toll immune pathway); Dome and Hop (JAK/STAT pathway); Dicer and R2D2 (RNAi pathway factors); Milt and Cnb (not immune-related genes). **(B)** Expression of anti-microbial peptides (AMPs) (DefA, DefC, DefD, and CecN) as controlled by immune pathways. Error bars represent standard error of mean (SEM). Comparisons used Student's *T*-TEST.

In the transgenic mosquito midguts, several components of the Toll and JAK/STAT pathways, namely Toll, Dome, and Hop, were significantly upregulated at day 3 post-infection (*t*-test; *p* = 0.0009, 0.0424, 0.0417, respectively; Figure 5A). Other components of the Toll and JAK/STAT pathways were upregulated as well (Supplementary Figure 2). Two components of the RNAi pathway, Dicer2 and R2D2, were also significantly upregulated at the same time point (*t*-test; *p* = 0.0045, 0.0218, respectively; Figure 5A) as compared to WT mosquitoes. Milton (Milt) and Centrobin (Cnb), two non-immune related genes responsible for organelle localization and centriole activity, respectively, were not affected by either CLEC18A expression or DENV infection (Figure 5A).

To further investigate potential modulation of the immune pathways, we quantified potential changes in the expression of antimicrobial peptides (AMPs). We found that Cecropin N (CecN), Defencin A (DefA), Defencin C (DefC), and Defencin D (DefD) showed a significant increasement in expression in the transgenic line as compared to WT line (*t*-test; *p* = 0.0229, 0.0110, 0.0121, 0.0119, respectively; Figure 5B).

Collectively, these results indicate that CLEC18A-2xHA expression led to an increase in expression for a number of Toll, JAK/STAT, and RNAi pathway components, which seemingly resulted in the aforementioned reduction in DENV infection rates in transgenic line.

### Transgenic CLEC18A Mosquitoes Show an Altered Midgut Microbiome

Modulated expression of C-type lectins in *A. aegypti* may result in alterations to the midgut microbiota, which plays a significant role in determining DENV infectivity (28, 33). We therefore used microbiome next-generation sequencing (NGS) to investigate differences in the midgut microbiomes of CLEC18A-2xHA-expressing mosquitoes.

**Figure 6:**
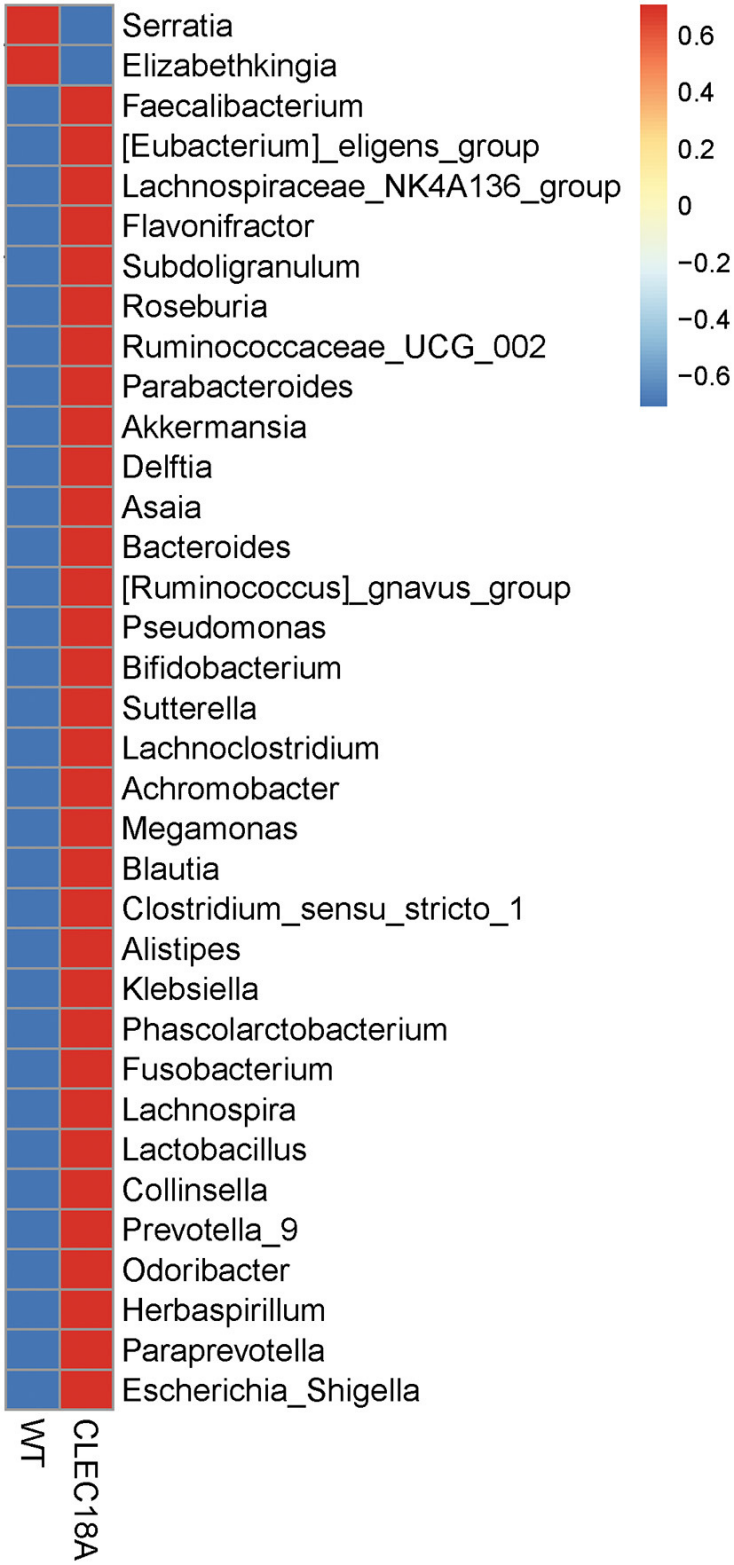
Fold change in midgut microbiome (genus) populations in CLEC18A transgenic mosquitoes. Twenty-five Midguts of WT and CLEC18A-2xHA transgenic mosquitoes were dissected for gut microbiome analysis. Results were analyzed and grouped by genus. Red indicates increased bacterial abundance as compared to the other test line.

Bacterial 16S rRNA was extracted from the midgut of these Pub promoter-driven CLEC18A-2xHA transgenic mosquitoes. We found significant changes in population distributions of major abundant bacterial families, such as *Enterobacteriaceae, Acetobacteraceae*, and *Weeksellaceae* in CLEC18A-2xHA expressing mosquitoes. In particular, two genera, *Elizabethkingia* and *Serratia*, were less abundant in these mutants, while *Herbaspirillum, Subdoligranulum*, and *Lactobacillus* were more abundant in the CLEC18A-2xHA transgenic line (Figure 6). These changes in mosquito midgut microbiota may result in corresponding changes to DENV infectivity.

These results demonstrate that CLEC18A-2xHA expression modulates both immune pathway expression levels and the midgut microbiome, resulting in protection from DENV infection.

## Discussion

The seemingly unstoppable rise of *A. aegypti*, and the severe diseases this vector can transmit, has led to an increased need for new methods of mosquito control. The generation of transgenic lines which block DENV infection offers one highly promising option for mosquito researchers. In order to explore mosquito immune responses to DENV infection, we expressed a human innate immune gene in a disease-transmitting vector for the first time. We found that transgenic expression of CLEC18A resulted in an enhancement of innate immunity pathways, potentially analogous to the role of CLEC18 in human immune signaling pathways.

In human cell lines, CLEC18A stimulated Interferon-β1 expression following DENV infection (unpublished results of Yun-Ting Tsou), suggesting that CLEC18A may be a factor in PRR-mediated cytokine secretion and interferon-stimulating gene activation during viral infection (47). IFN-β expression in human dendritic cells and macrophages is controlled by a Toll-like signaling pathway (57) similar to the mosquito Toll pathway. A mouse model has also demonstrated that human CLEC18A associates with TLR3 and dsRNA in endosomes to enhance TLR3-mediated IFN expression (49). In mosquitoes, CLEC18A may also associate with a Toll or DENV/Toll receptor to enhance innate immune signaling cascades and AMPs expression.

The amino acid sequences of the CTL domains from the three human CLEC18 protein isoforms are highly similar. Isoform CLEC18B is predicted to have nine extra amino acids, LVWLSAAMG, located between positions 464 and 465 of the CLEC18A CTLD (47). When testing interactions between CLEC18 and dengue virus particle, the extra amino acids of CLEC18B affected its interaction with DENV (Figure 1B). These extra amino acids in the CTLD of CLEC18B also abolished the binding ability of CTLD to F3 polysaccharides isolated from medicinal fungi *Ganoderma lucidum* (GLPS-F3) (47), which contain abundant polysaccharides such as glucose, mannose, fructose, galactose, GlcNAc xylose, and rhamnose (47, 58). Compared to CLEC18A, the CTLD of CLEC18C has a single variation, D421N (47). The presence of D421N seemed to reduce CLEC18C interactions with prM-E and virus particles (Figure 1), suggesting this amino acid plays an important role in this interaction.

There are no apparent mosquito orthologs of human CLEC18A. We have listed three mosquito CTLs that have similar domain architecture to CLEC18A in Supplementary Figure 1A, which we identified following a phylogenic analysis focusing on the CTLDs of 48 *A. aegypti* C-type lectins [the information of four C-type lectins were combined/removed in AaegL5.2 geneset in Vectorbase when compared to (26)] and human CLEC18A to find closely related mosquito C-type lectins to CLEC18A (Supplementary Figure 1B). This analysis showed that the CTLDs of two mosquito C-type lectin proteins, AAEL014357 and AAEL021200, were related to CLEC18A (Supplementary Figure 1C). However, our results show that the transcripts of AAEL014357 and AAEL021200 were barely detected in the mosquito midgut (Ct value > 32 in both WT and CLEC18A-2xHA transgenic line), which agrees with the results of a previous study (26).

The expression of mosquito-specific C-type lectins can influence DENV infection characteristics and success rates both directly and indirectly. For example, AAEL008069 expression decreases DENV replication rates (59); on the other hand expression of other C-type lectins, such as mosGCTL-3, mosGCTL-15, and mosGCTL-19, can increase DENV replication (32, 43).

Mosquito infection with DENV (or other arboviruses) triggers an innate immune response which limits viral replication in order to prevent mortality. The RNAi response pathway plays a major role in this response to virus infection, with mosquitoes generating siRNAs following infection. Depletion of the RNAi pathway via silencing of Dcr2 or R2d2 results in a significant increase in viral titer (60). JAK/STAT and Toll pathways are also involved in DENV infection responses, where silencing of pathway components, for example via knockdown of Rel1, Dome, or Hop, results in an increase in viral load (42, 61).

We found upregulation of several components of these innate immune pathways in the CLEC18A-2xHA-expressing line. The expression of CLEC18A-2xHA in the mosquito may therefore enhance anti-DENV activity following infection and result in a reduction in DENV viral load. These immune response pathways can be influenced via multiple signaling molecules. Our findings reveal no significant differences between CLEC18A-2xHA line and controls in the expression level of Vago, which is induced by the IMD pathway, suggesting that CLEC18A activation through the JAK/STAT pathway may act independently of Vago. Associations between the IMD pathway and DENV infection remain unclear. While one study found downregulation of IKK2, a factor of the IMD pathway, following DENV infection (42), other reports indicate that silencing of Caspar, an inhibitor of the IMD pathway, does not affect viral load (42, 62). This stands in direct contrast to reports that direct silencing of the IMD pathway seems to increase viral load (62).

Multiple AMPs are induced by innate immune signaling pathways following DENV infection, such as *defensin* (Def), *cecropin* (Cec), *gambicin*, and *attacin* (59). Here we found increased expression of DefA, DefC, DefD, and CecN when CLEC18A-2xHA was expressed in the mosquito. These four AMPs have been found to be elevated in DENV-2 infected *A. aegypti*, suggesting a key role in the immune response for these specific components (59). Depletion of these AMPs (apart from DefA) via gene silencing also significantly enhances the DENV2 viral load in *A. aegypti* (59).

Here we normalized the expression level of all immune components with *A. aegypti* RPS7, a commonly used normalizer. However, other “housekeeping” genes, such as actin, α-tubulin, and RPS17, have also been validated for their usability as qPCR normalizers (54). We therefore analyzed the expression profile of RPS7 when normalized by actin and α-tubulin to confirm its' suitability. We found no significant differences in RPS7 expression (*p* > 0.05 for all comparisons) when comparing days 1, 3, and 7 post-infection in WT or CLEC18A-2xHA transgenic lines (Supplementary Figure 3, right panel). RPS7 thus appears a suitable normalizer given that expression remained relatively stable across all designated experimental time points.

In addition, C-type lectins can significantly influence the balance of bacterial populations in the mosquito midgut (28). Previous reports have indicated that the presence of *Serratia marcescens* is crucial for DENV infection (33). Enhancin, secreted by *S. marcescens*, digests mucins on the midgut epithelia and facilitates DENV infection, where mosquitoes provided with antibiotics showed a significant decrease in DENV infectivity. An imbalance of microbiota in the mosquito midgut has also been correlated with changes in the expression of immune system genes related to the Toll pathway and several AMPs (42). Moreover, introducing the *Wolbachia* wAlbB strain into *A. aegypti* induces the production of reactive oxygen species (ROS), which leads to the activation of the Toll pathway to control DENV replication in mosquitoes (63). Since CLEC18A expression in mosquito C6/36 cell lines did not abolish its secretory ability (unpublished data of Feng-Shuo Pai), our NGS analyses showed that the reduction in the abundance of members of the *Serratia* genus in the midguts of CLEC18A-2xHA expressing line may be due to secreted CLEC18A-2xHA directly or indirectly, and may thus result in a decrease of DENV viral load.

Taken together, our results show that transgenic expression of a human C-type lectin, CLEC18A-2xHA, decreased DENV infectivity. CLEC18A was able to associate with DENV viral proteins and virus particles, and potentially acts similarly to PRRs involved in innate immune system processes. CLEC18A-2xHA enhanced innate immune pathways and upregulated the expression of AMPs, providing an important mechanism for the prevention of DENV infection. Expression of CLEC18A also affected the microbiome balance in the midgut, potentially decreasing bacterial populations that facilitate DENV infection. In humans, CLEC18A is a secretory protein that can be detected in the bloodstream and may be a potential biomarker of HBV infection (48). Except CLEC18A, other human C-type lectin proteins that were reported to interact with DENV, such as DC-SIGN or CLEC5A could be used to investigate the potential in mosquito's innate immune response. New methods of vector control are desperately needed to combat the spread of DENV. The use of transgenic mosquitoes capable of inhibiting DENV replication offers substantial promise.

## Data Availability Statement

The original contributions generated for the study are included in the article/Supplementary Material, further inquiries can be directed to the corresponding author/s.

## Author Contributions

LC, W-LL, J-JT, and C-HChe: conception and design. LC and MS: formal analysis. LC, W-LL, Y-TT, C-HChi, and S-CW: investigation and data collection. LC, J-CL, K-LL, and Y-LH: methodology. C-HChe: funding. LC, W-LL, S-LH, and C-HChe: writing – original draft. All authors contributed to the article and approved the submitted version.

## Conflict of Interest

The authors declare that the research was conducted in the absence of any commercial or financial relationships that could be construed as a potential conflict of interest.
