# Supplementary material for: Transgenic Expression of Human C-Type Lectin Protein CLEC18A Reduces Dengue Virus Type 2 Infectivity in Aedes aegypti
Source: Front Immunol. 2021 Mar 9;12:640367. doi: 10.3389/fimmu.2021.640367 (PMC7985527; doi:10.3389/fimmu.2021.640367)

SUPPLEMENTARY INFORMATION

MATERIAL AND METHODS

Phylogenic analysis

CTL domain (CTLD) sequences of 48 *A. aegypti* C-type lectin proteins were collected from the Vectorbase (AaegL5.2 geneset of Vectorbase) followed the reference. CTLD of these mosquito C-type lectin proteins and human CLEC18A were aligned by using MUSCLE with parameter: -2.9 gap open and -0.2 gap extend penalties in MEGA X, followed by built a tree with Neighbor-Joining method by using MEGA X (pairwise deletion option).

FIGURE LEGENDS

Supplementary Figure 1. Phylogenic analysis of human CLEC18A and *A. aegypti* C-type lectin proteins. (A) Mosquito C-type lectins with similar domain structures to CLEC18A. **(B)** Evolutionary history was inferred using the Neighbor-Joining method. The tree is drawn to scale, with branch lengths in the same units as those of the evolutionary distances used to infer the phylogenetic tree. The evolutionary distances were computed using the number of differences method and are in units of the number of amino acid differences per sequence. The analysis involved 49 amino acid sequences and conducted in MEGA X. **(C)** The two mosquito C-type lectin proteins that were related to human CLEC18A, AAEL014357 and AAEL021200 are listed together with their structures. EGF and EGF-like domains of CLEC18A are shown in orange blocks. Transmembrane domain (TM) of mosquito CTL proteins are shown in yellow blocks.

Supplementary Figure 2. Expression levels of the components of immune pathways following oral challenge with DENV2. WT and CLEC18A-2xHA transgenic mosquitoes were infected with DENV via exposure to an artificial blood meal. The midguts of pre-BM, 1, 3, and 7 days post-infection mosquitoes were analyzed to determine the relative expression of components from the immune pathways with qPCR analysis. The expression of Rel1A and Cactus (Toll immune pathway); Imd, Rel2, Caspar, and Vago (Imd pathway); STAT and PIAS (JAK/STAT pathway). Error bars represent standard error of mean (SEM). Comparisons used Student's T-TEST.

Supplementary Figure 3. Expression profile of RPS7 in mosquito midgut. WT and CLEC18A-2xHA transgenic mosquitoes were infected with DENV via exposure to an artificial blood meal. The midguts of pre-BM, 1, 3, and 7 days post-infection mosquitoes were analyzed to determine the relative expression of RPS7 via qPCR analysis. Expression levels of RPS7 were normalized with either actin (upper panel) or

α-tubulin (lower panel). Left panels show comparisons prior to infection, while right panels include comparisons 1, 3 and 7 days post-infection. Error bars represent standard error of mean (SEM). Student t-tests were used for all comparisons, with a significance level set at 0.05. n.s. = non-significant.

Supplementary Figure 1A

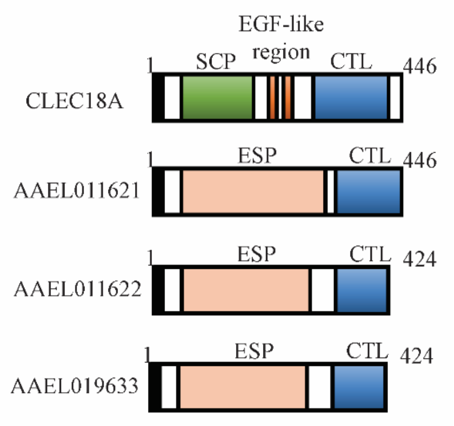

Supplementary Figure 1B

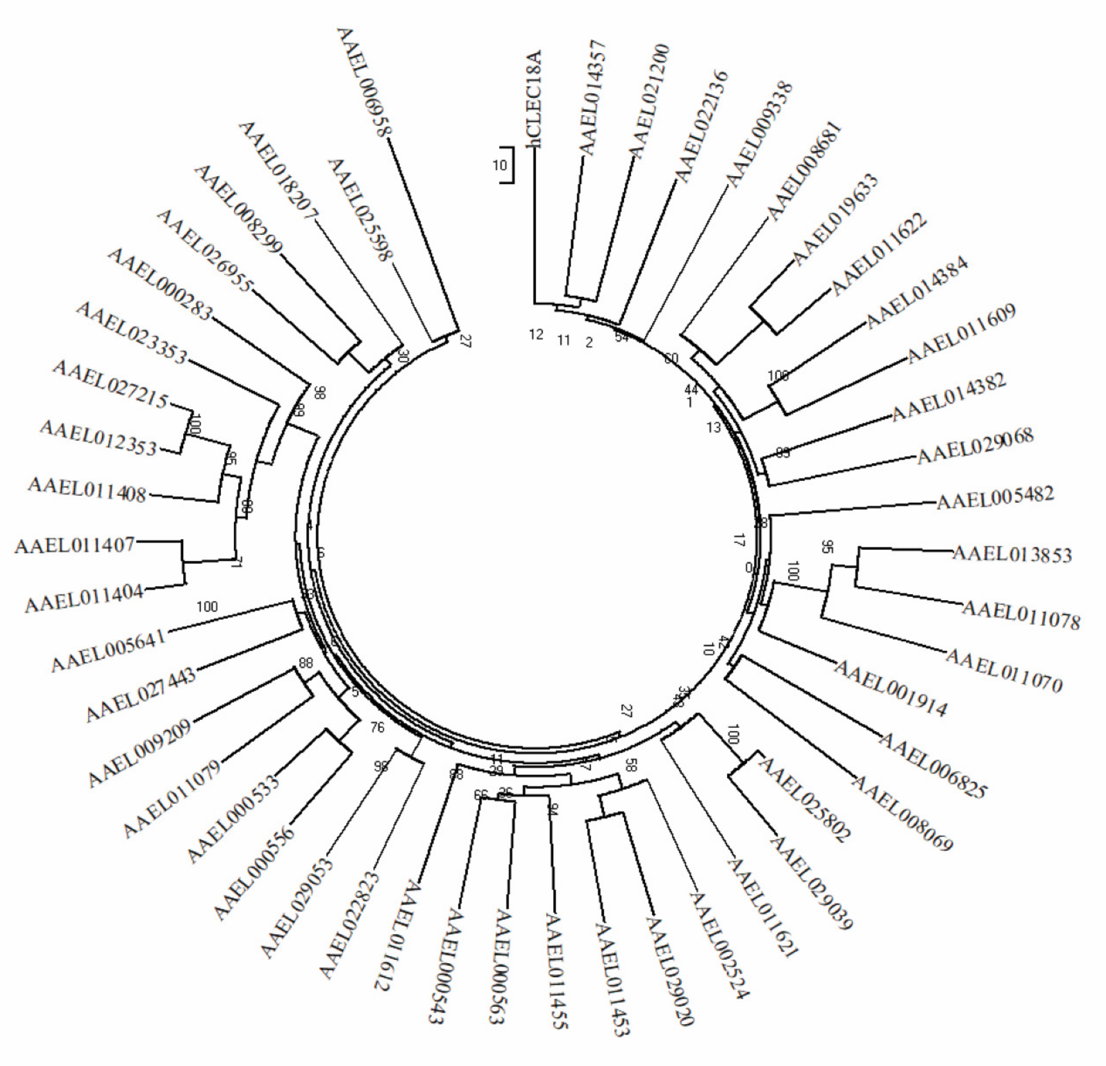

Supplementary Figure 1C

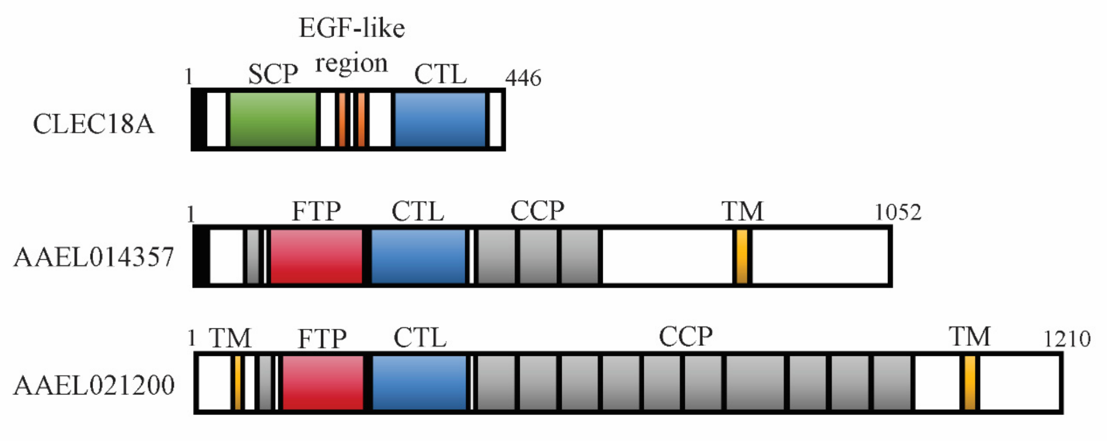

Supplementary Figure 2

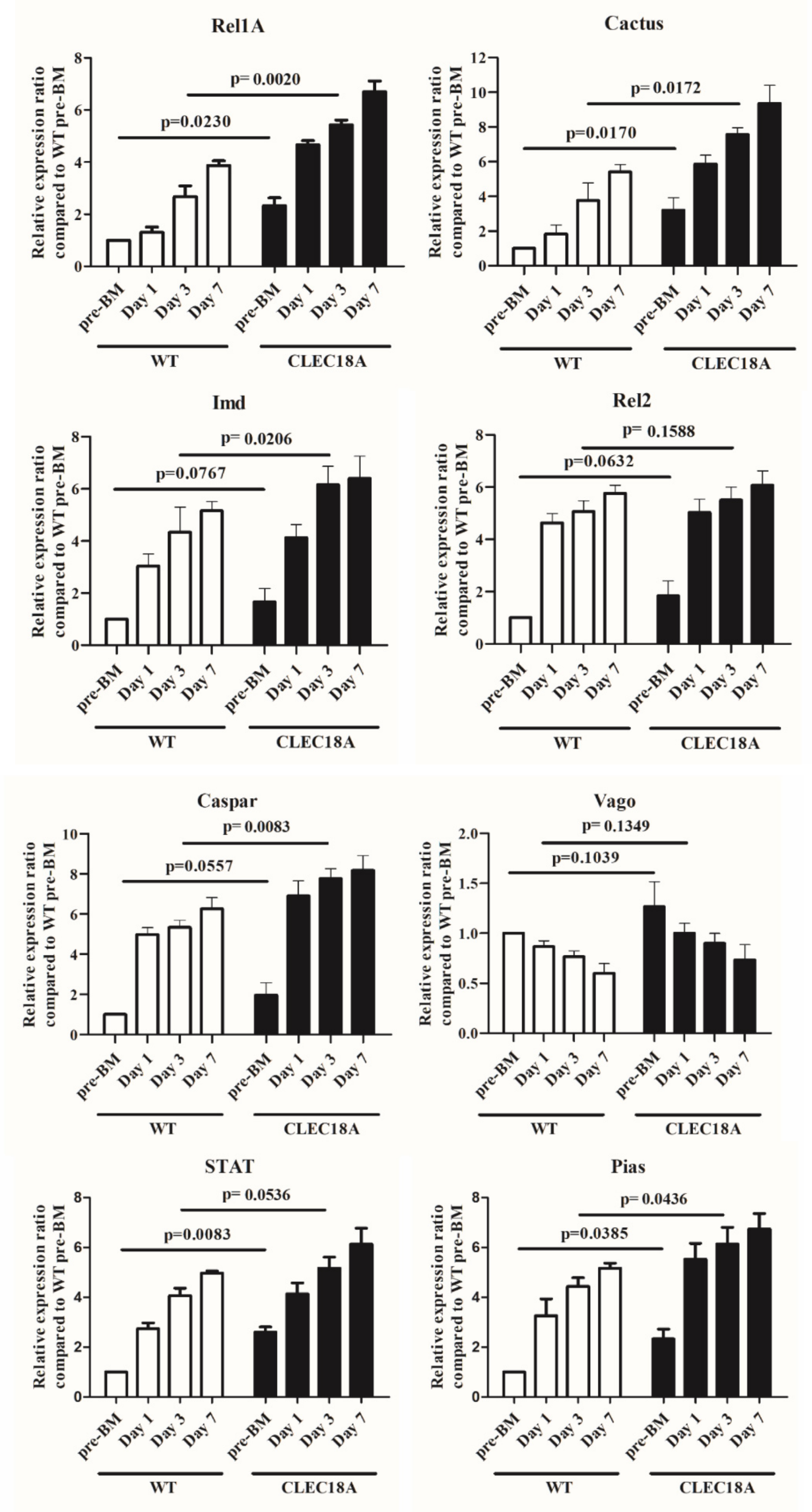

Supplementary Figure 3

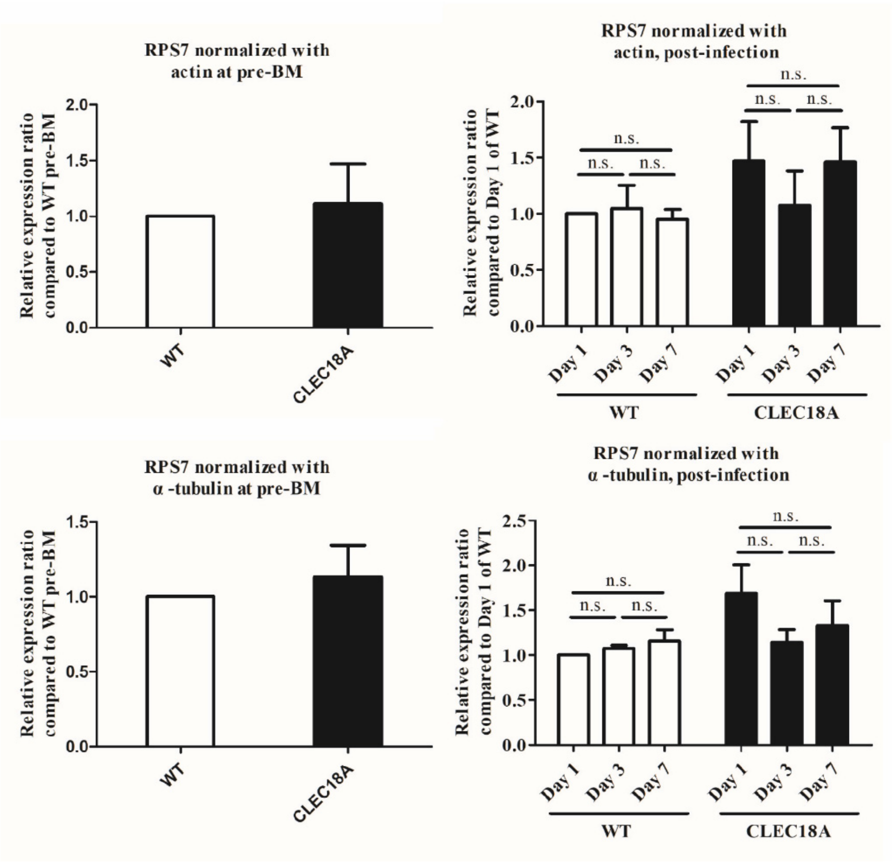

Supplement: Supplementary file 1 [file Data_Sheet_1.pdf]
